# The Gene Variants of Maternal/Fetal Renin-Angiotensin System in Preeclampsia: A Hybrid Case-Parent/Mother-Control Study

**DOI:** 10.1038/s41598-017-05411-z

**Published:** 2017-07-11

**Authors:** Heng Zhang, Ying-Xue Li, Wei-Jun Peng, Zhi-Wei Li, Chun-Hua Zhang, Hai-Hong Di, Xian-Ping Shen, Jun-Feng Zhu, Wei-Rong Yan

**Affiliations:** 1 Department of Epidemiology and Biostatistics, School of Public Health, Tongji Medical College of Huazhong University of Science & Technology, Wuhan, 430030 China; 2 Department of Medical Services, Luoyang Maternal and Child Health Care Hospital, Luoyang, 471000 China; 3 Department of Obstetrics and Gynecology, Anyang Maternal and Child Health Care Hospital, Anyang, 455000 China; 4 Department of Administration, Yichang Maternal and Child Health Care Hospital, Yichang, 443000 China; 5 Department of Medical Services, Yichang Maternal and Child Health Care Hospital, Yichang, 443000 China; 6 Department of Obstetrics and Gynecology, Yichang Maternal and Child Health Care Hospital, Yichang, 443000 China

## Abstract

Preeclampsia (PE) is a common pregnancy-related complication, and polymorphisms in angiotensinogen (*AGT*), angiotensin-converting enzyme (*ACE*), and angiotensin II type 1 receptor (*AT1R*) are believed to contribute to PE development. We implemented a hybrid study to investigate the influence of maternal and fetal *ACE* I/D, *ACE* G2350A, *AGT* M235T, *AGT* T174M, and *AT1R* A1166C polymorphisms on PE in Han Chinese women. Polymorphisms were genotyped in 1,488 subjects (256 patients experiencing PE, along with their fetuses and partners, and 360 normotensive controls with their fetuses). Transmission disequilibrium tests revealed that *ACE* I/D (*P* = 0.041), *ACE* G2350A (*P* = 0.035), and *AT1R* A1166C (*P* = 0.018) were associated with maternal PE. The log-linear analyses revealed that mothers whose offspring carried the MM genotype of *AGT* M235T had a higher risk of PE (OR = 1.54, *P* = 0.010), whereas mothers whose offspring carried the II genotype of *ACE* I/D or the GG genotype of *ACE* G2350A had a reduced risk (OR = 0.58, *P* = 0.039; OR = 0.47, *P* = 0.045, respectively). Our findings demonstrate that fetal *ACE* I/D, *ACE* G2350A, *AGT* M235T, and *AT1R* A1166C polymorphisms may play significant roles in PE development among pregnant Han Chinese women.

## Introduction

Preeclampsia (PE) is a common pregnancy-related complication and a major contributor to maternal and infant morbidity and mortality^1, 2^. Women with PE and their infants are at an increased risk of cardiovascular and renal disease, and type 2 diabetes in later life^3, 4^. Furthermore, the risk of low birth weight, neonatal asphyxia, and perinatal fetal death are significantly higher in these babies^5^. PE complicates approximately 2–8% of pregnancies worldwide^2^, with an incidence of 5.22% in China^6^. Despite extensive researches, the detailed pathogenesis of PE remains unclear.

The renin-angiotensin system (RAS) is a peptide cascade comprising the following proteins: rennin (REN), angiotensinogen (AGT), angiotensin-converting enzyme (ACE), and angiotensin I, II (ANG I, ANG II), and 1–7 (ANG1–7), as well as angiotensin II type 1 receptor (AT1R) and angiotensin II type 2 receptor (AT2R); RAS is believed to contribute to PE development^7–9^. All RAS components are expressed in and around the spiral arteries in pregnant women during the first trimester, where they contribute to pregnancy-induced vessel remodeling^10, 11^. In addition to circulating RAS, a tissue-based RAS exists in the utero-placental unit^7^. This local RAS influences the regulation of regional maternal intervillous blood flow and assists in local spiral artery remodeling, and its dysregulation causes shallow placental implantation and utero-placental ischemia^12, 13^, leading to PE^14^. Retrospective studies have suggested that heritable allelic variations, particularly those in the utero-placental RAS, are associated with defective placental vascular development. Analysis of these variations could become the cornerstone for understanding the genetics of PE^15^. Furthermore, the placenta consists of both maternal and fetal tissue, and placental RAS components are partly derived from the expression of the fetal genes^16–18^. Therefore, both maternal and fetal RAS genes might contribute to PE pathogenesis. A study based on the Swedish Birth Registry revealed that 35% and 20% of the variance in PE liability is attributable to maternal and fetal genetic effects, respectively^19^. However, few studies have considered the maternal and fetal effects of RAS gene polymorphisms together.

Among the RAS gene single nucleotide polymorphisms (SNPs), *ACE* insertion/deletion (*ACE* I/D, rs1799752), *ACE* G2350A (rs4343), *AGT* Met235Thr (M235T, rs699), *AGT* Thr174Met (T174M, rs4762), and AT1R A1166C (rs5186) are related to tissue and plasma RAS component concentrations, and therefore thought to be relevant to PE development^20–25^. Many noninfectious diseases are caused by low-penetrance alleles interacting with environmental factors^26^. A series of studies have revealed the gene–environment interactions between maternal RAS SNPs and environmental factors such as cigarette smoking, BMI, mental stress^27–29^. Therefore, the objective of the current study was to investigate the effects of these maternal and fetal polymorphisms, as well as potential maternal gene–environment interactions, on PE.

## Results

### Patient Characteristics

The characteristics of the study participants are presented in Table 1. There was no difference in maternal age between the two groups. The systolic and diastolic BP values were much higher in PE patients than in controls (both *P* < 0.001). Compared with the control group, a higher proportion of patients were educated to below high school level (61.8% *vs*. 46.6%, *P* < 0.001), and had a family history of hypertension (28.9% *vs*. 18.8%, *P* = 0.006). Pre-pregnancy body mass index (BMI) was significantly higher (21.36 ± 3.59 kg/m^2^ *vs*. 23.06 ± 3.78 kg/m^2^, *P* < 0.001), and gestational age at delivery was significantly lower (37.29 ± 2.47 weeks *vs*. 38.46 ± 2.48 weeks, *P* < 0.001) in patients than in controls. The average fetal birth weight was lower in patients than in controls (2.97 ± 0.68 kg *vs*. 3.27 ± 0.44 kg, *P* < 0.001), and the PE group had a higher proportion of intrauterine growth restriction (IUGR) cases than the control group (27.5% *vs*. 3.9%, *P* < 0.001).

**Table 1:** Relationship between risk factors and preeclampsia (PE).

	Case $\overline{{\bf{x}}}$ ± SD/n (%)	Control $\overline{{\bf{x}}}$ ± SD/n (%)	*P*
Maternal age (year)	28.77 ± 5.39	28.33 ± 4.86	0.298
Systolic BP (mm Hg)	150.90 ± 14.78	112.24 ± 11.57	**<0.001**
Diastolic BP (mm Hg)	101.18 ± 10.12	72.11 ± 8.06	**<0.001**
Education background			**<0.001**
Junior middle school and Lower	154 (61.8%)	159 (46.6%)	
High school or above	95 (38.2%)	182 (53.4%)	
Pre-pregnancy BMI (Kg/m^2^)	23.06 ± 3.78	21.36 ± 3.59	**<0.001**
Parity			0.074
Primipara	132 (52.0%)	204 (59.3%)	
Multipara	122 (48.0%)	140 (40.74%)	
Fetal birth weight	2.97 ± 0.68	3.27 ± 0.44	**<0.001**
IUGR			
Yes	63 (27.5%)	12 (3.9%)	**<0.001**
No	166 (72.5%)	293 (96.1%)	
Gestational age at delivery (week)	37.29 ± 2.47	38.46 ± 2.48	**<0.001**
Family history of hypertension			**0.006**
Yes	67 (28.9%)	59 (18.8%)	
No	165 (71.1%)	255 (81.2%)	
Smoking during pregnancy			0.705
Yes	2 (0.8%)	5 (1.4%)	
No	248 (99.2%)	340 (98.6%)	

SD: standard deviation; BMI: body mass index; bold value indicates significant result; IUGR: intrauterine growth restriction.

### SNP Genotype Distributions

The association analyses of *ACE* I/D, *ACE* G2350A, *AGT* M235T, *AGT* T174M, and *AT1R* A1166C were performed separately. For each SNP, we excluded families in which one or more members failed to be genotyped, as well as those inconsistent with Mendelian inheritance. The distribution of genotypes and alleles of all five polymorphisms were in Hardy-Weinberg Equilibrium (HWE). The genotype and allele counts of the five SNPs are shown in Supplementary Table S1. The associations of the polymorphisms with PE are shown in Table 2. The fetal *AGT* M235T polymorphism in the dominant model (TT + MT/MM) was found to be associated with PE (OR = 1.40, 95% CI: 1.01–1.94, *P* = 0.043). However, the association disappeared after multiple testing corrections (false discovery rate [FDR]).

**Table 2:** Single locus analysis for all the single nucleotide polymorphisms (SNPs) genotyped

SNP	Model	Maternal			Fetal		
		OR	*P*	P*	OR	*P*	*P* ^*^
*ACE* I/D	dominant	1.04 (0.73–1.48)	0.822	0.997	0.84 (0.60–1.19)	0.327	0.997
	recessive	0.89 (0.60-1.33)	0.578	0.997	0.87 (0.56–1.33)	0.516	0.997
*ACE* G2350A	dominant	1.11 (0.79–1.55)	0.368	0.997	0.88 (0.63–1.22)	0.442	0.997
	recessive	0.86 (0.52–1.43)	0.559	0.997	0.72 (0.40–1.30)	0.274	0.997
*AGT* M235T	dominant	1.10 (0.79–1.53)	0.651	0.997	1.40 (1.01–1.94)	**0.043**	0.930
	recessive	0.82 (0.45–1.51)	0.455	0.997	0.98 (0.53–1.80)	0.946	1.000
*AGT* T174M	dominant	1.00 (0.66–1.50)	0.781	0.997	0.79 (0.51–1.23)	0.500	0.997
	recessive	1.85 (0.49–6.96)	0.355	0.997	5.95 (0.66–53.56)	0.178	0.997
*AT1R* A1166C	dominant	-	0.505	0.997	-	0.600	0.997
	recessive	1.05 (0.63–1.74)	1.000^*^	1.000	0.95 (0.54–1.67)	1.000^*^	1.000

Statistical significance was tested at *P* < 0.05; OR, odds ratio; CI, confidence interval; *, using Fisher exact probability; “-” means the result could not be calculated because of limited counts; *P* ^*^, corrected *P*-value after FDR procedure.

### Transmission Disequilibrium Test (TDT) Analyses in Case-Parent Triad Families

TDTs were employed to test the linkage between the polymorphisms and PE, and to exclude the false association caused by the population structure. We selected all the heterozygous parents, and identified the alleles of the five SNPs that were transmitted or untransmitted from the parents to the offspring. At *P* = 0.05, significant results were obtained for *ACE* I/D, *ACE* G2350A, and *AT1R* A1166C, suggesting that these three fetal SNPs are associated with PE (Table 3).

**Table 3:** Results of transmission disequilibrium test (TDT) analyses of the SNPs in case-parent triad families.

SNP	counts of trans *vs*. non-trans alleles			TDT	*P*
	transmitted allele	non-transmitted allele			
		A1	A2		
*ACE* I/D	A1	160	139		
	A2	107	94	4.16	0.041
*ACE* G2350A	A1	201	124		
	A2	93	50	4.43	0.035
*AGT* M235T	A1	265	84		
	A2	108	41	3.00	0.083
*AGT* T174M	A1	395	40		
	A2	34	7	0.49	0.486
*AT1R* A1166C	A1	410	38		
	A2	20	2	5.59	0.018

A1 and A2 represent the two alleles of each SNP.

### Estimation of the Effects of Maternal and Fetal Polymorphisms

To estimate the effects of the maternal and fetal genes, a log-linear model was employed. As shown in Table 4, three fetal SNPs were found to be related to PE. Mothers of offspring carrying the *ACE* I/D II or *ACE* G2350A GG genotypes had a reduced PE risk (OR = 0.58, 95% CI: 0.36–0.91; OR = 0.47, 95% CI: 0.26–0.88, respectively). Mothers of offspring carrying the *AGT* M235T MM genotype had an increased risk of developing PE (OR = 1.54, 95% CI: 1.13–2.10). No significant associations were detected between the five maternal SNPs, as well as fetal *AGT* T174M and *AT1R* A1166C polymorphisms, and PE. Furthermore, the effects of these SNPs on severe (s)PE and mild (m)PE were analyzed (Table 5). An increased risk of sPE was observed for the fetal *AGT* M235T MM genotype. Neither the fetal SNPs nor the maternal SNPs were associated with mPE significantly.

**Table 4:** Genetic association of the maternal and fetal genotypes (SNPs in *ACE*, *AGT*, and *AT1R*) with PE.

SNP	Genotype	Maternal genotype		Offspring genotype	
		OR (95% CI)	*P*	OR (95% CI)	*P*
*ACE* I/D	DD	reference	0.491	reference	0.039
	ID	1.12 (0.79–1.59)		0.80 (0.64–1.22)	
	II	1.31 (0.83–2.04)		**0.58 (0.36–0.91)**	
*ACE* G2350A	AA	reference	0.589	reference	0.045
	AG	0.99 (0.67–1.46)		0.86 (0.61–1.21)	
	GG	1.19 (0.63–2.26)		**0.47 (0.26–0.88)**	
*AGT* M235T	TT	reference	0.570	reference	0.010
	MT	0.97 (0.70–1.34)		1.24 (0.70–2.23)	
	MM	0.70 (0.39–1.27)		**1.54 (1.13–2.10)**	
*AGT* T174M	TT	reference	0.085	Reference	0.442
	MT	1.68 (0.99–2.82)		0.76 (0.46–1.26)	
	MM	3.16 (0.60–16.78)		1.35 (0.33–5.55)	
*AT1R* A1166C	AA	reference	0.699	Reference	0.364
	AC	1.17 (0.73–1.89)		0.69 (0.41–1.15)	
	CC	2.05 (0.17–24.31)		-	

“-” means the result could not be calculated because of limited counts.

**Table 5:** Genetic association of the maternal and fetal genotypes (SNPs in *ACE*, *AGT*, and *AT1R*) with mild (m)PE and severe (s)PE.

SNP	Genotype		Maternal genotype		Offspring genotype	
			OR (95% CI)	*P*	OR (95% CI)	*P*
*ACE* I/D	DD		reference		reference	
	ID	m	1.08 (0.64–1.89)	0.491	0.43 (0.18–1.03)	0.100
	II		1.91 (0.32–11.44)		0.27 (0.06–1.17)	
	ID	s	1.07 (0.66–1.74)	0.654	1.14 (0.76–1.71)	0.054
	II		1.31 (0.71–2.43)		0.65 (0.36–1.16)	
*ACE* G2350A	AA		reference		reference	
	AG	m	0.96 (0.45–2.07)	0.260	0.52 (0.23–1.15)	0.215
	GG		2.44 (0.75-8.01)		0.39 (0.09–1.68)	
	AG	s	0.95 (0.81–1.63)	0.700	0.91 (0.65–1.27)	0.354
	GG		1.16 (0.65–2.05)		0.64 (0.36–1.17)	
*AGT* M235T	TT		reference		reference	
	MT	m	0.74 (0.31–1.77)	0.508	1.32 (0.31–1.74)	0.477
	MM		0.45 (0.39–5.44)		1.47 (0.39–5.52)	
	MT	s	0.94 (0.67–1.33)	0.437	1.19 (0.62–2.30)	0.004
	MM		0.66 (0.35–1.32)		**1.74 (1.25–2.41)**	
*AGT* T174M	TT		reference		reference	
	MT	m	2.00 (0.18–22.04)	0.085	1.50 (0.42–5.31)	0.442
	MM		2.33 (0.60–9.02)		-	
	MT	s	1.60 (0.90–2.84)	0.174	0.67 (0.39–1.16)	0.283
	MM		4.31 (0.43–43.69)		1.22 (0.29–5.11)	
*AT1R* A1166C	AA		reference		reference	
	AC	m	1.17 (0.21–2.83)	0.928	0.76 (0.21–2.80)	0.921
	CC		-		-	
	AC	s	1.26 (0.76–2.07)	0.545	0.67 (0.38–1.16)	0.361
	CC		2.46 (0.21–29.49)		-	

“-” means the result could not be calculated because of limited counts.

### Maternal Gene-Environment Interaction Analyses

A likelihood ratio test (LRT) based on the log-linear approach was used to evaluate the interaction between environmental factors and maternal genes. In the log-linear model of LRT, the patients are separated into exposed and unexposed groups ^26^. Therefore, in the current study, only the two significant categorical variables, education background (beneath or above high school) and pre-pregnancy BMI (<24 kg/m^2^ or ≥24 kg/m^2^) were evaluated. The results revealed that pre-pregnancy BMI had significant interactions with *AGT* M235T (*P*_LRT_ = 0.029) and *AGT* T174M (*P*_LRT_ = 0.015; Table 6). Then, we calculated the ORs for each maternal genotype in the two strata of BMI (Table 7). Among women with BMI ≥ 24 kg/m^2^, the ORs for the maternal M allele of both the SNPs were <1, but without statistical significance (*P* > 0.05).

**Table 6:** LRTs for maternal gene-environment interaction

SNP	Environmental factor	χ^2^	*P*_LRT_
*ACE* I/D	education background	0.0159	0.992
	pre-pregnancy BMI	4.8175	0.09
*ACE* G2350A	education background	0.042	0.979
	pre-pregnancy BMI	0.682	0.711
*AGT* M235T	education background	5.66	0.059
	pre-pregnancy BMI	7.07	**0.029**
*AGT* T174M	education background	0.428	0.807
	pre-pregnancy BMI	8.428	**0.015**
*AT1R* A1166C	education background	0.00152	0.999
	pre-pregnancy BMI	0.152	0.927

LRT, likelihood ratio test; the tests were conducted using case-parent triads.

**Table 7:** Genetic association results for the maternal *AT1R* C573T genotypes within different strata of pre-pregnancy BMI.

SNP	BMI (n)	Genotype	OR (95% CI)	*P*
*AGT* M235T	<24 kg/m^2^ (137)	TT	reference	0.352
		MT	1.02 (0.67–1.55)	
		MM	0.54 (0.22–1.29)	
	≥24 kg/m^2^ (80)	TT	reference	0.880
		MT	0.96 (0.52–1.74)	
		MM	0.76 (0.26–2.24)	
*AGT* T174M	<24 kg/m^2^ (134)	TT	reference	0.339
		MT	1.42 (0.79–2.54)	
		MM	2.51 (0.68–9.31)	
	≥24 kg/m^2^ (75)	TT	reference	0.473
		MT	0.55 (0.21–1.45)	
		MM	0.86 (0.12–5.96)	

## Discussion

To our knowledge, this is the first study to investigate the association between maternal/fetal RAS polymorphisms and PE in a hybrid design with a large sample size of 1,488 patients, focusing on the Chinese Han population.

PE is believed to be a two-stage disease triggered by abnormal placentation and deficient spiral artery remodeling, progressing with placental ischemia, and the latter stage leads to the development of hypertension and proteinuria^30^. The widespread vascular and endothelial dysfunction during placental ischemia may be caused by an imbalance of angiogenic factors, including RAS components^31^. A few studies had previously explored the association between RAS genes and PE^32–38^, but they mainly used a case-control study design. It must be noted here that the case-control study design is less sensitive in the detection of parent-of-origin genetic effects. Moreover, the test power is lower than that of family-based designs such as the case-parent/mother-control design^39, 40^. Therefore, to elucidate the roles of maternal and fetal RAS genes in PE development, we adopted a hybrid case-parent/mother-control design, instead of a traditional case-control study design.

In the current study, allele and genotype frequencies were investigated for all five RAS genes (*ACE* I/D, *ACE* G2350A, *AGT* M235T, *AGT* T174M, and *AT1R* A1166C); however, no significant differences were observed in the patients or fetuses. The TDT and mating-type–stratified likelihood-based methods (log-linear approach) applied in the current study can overcome the limitations of the case-control study design, as it involves the comparison of the genotypes of cases to that of their nuclear family members, whose nontransmitted chromosomes serve as ethnically matched genetic controls^41^. In the TDT, positive results were obtained for *ACE* I/D, *ACE* G2350A, and *AT1R* A1166C. The results of the log-linear modeling approach were approximately consistent with those of the TDT. Compared with the TDT results, the log-linear modeling approach failed to detect the effect of fetal *AT1R* A1166C, but detected a correlation between fetal *AGT* M235T and PE. In the log-linear modeling approach, there was no count for the CC type of fetal *AT1R* A1166C polymorphism, and therefore, we could not calculate its effect. It may be noted here that the power of log-linear modeling in a hybrid design with the current sample size can approach 0.9. However, the TDT resulted in a power of 0.72, which might explain why the log-linear modeling approach could find a positive association between fetal *AGT* M235T and PE, whereas TDT could not. Comprised of population-based controls and affected family-triads, the hybrid design affords a more detailed analysis of the genetic effects of the offspring genes^42^, and is particularly suitable for maternal- and offspring-based genetic analyses of gestational and congenital diseases. It is more powerful than a case-control design^43^ or pure case-parent design^40^ with the same sample size.

In addition, in this study, we investigated the effects of the five SNPs on sPE and mPE respectively. Since the sample of mPE (39) was limited, we failed to find any associations between the fetal/maternal SNPs with mPE. However, the positive effects of the fetal *ACE* I/D II type (*P* = 0.039) and fetal *ACE* G2350A GG type (*P* = 0.045) in PE vanished in sPE (*P*-value were 0.054 and 0.354 respectively) as the sample size of sPE diminished. In contrast, the most significant risk of PE observed for fetal *AGT* M235T MM type (*P* = 0.010) remained in sPE (*P* = 0.004). Meta-analyses, case–control studies and animal experiments have shown the AGT M235T polymorphism to be related to higher AGT levels and an increased risk of hypertension^12, 44^. The AGT M235T polymorphism might therefore be of higher functional relevance in modifying RAS activity.

Fetal-derived placental tissue participates in RAS factor secretion and regulation, thereby possibly contributing to PE development^8^. Previous molecular biology-based studies have provided clues on the risk-inducing effect of the *AGT* M235T MM genotype and the protective effects of the *ACE* I/D II and *ACE* G2350A GG genotypes. The expression of the I polymorphism of *ACE* I/D inhibits ACE activity in serum, and therefore, decreases the risk of PE, compared to the D polymorphism^45^. Another study has indicated that a combination of the AA genotype of *ACE* G2350A and DD genotype of *ACE* I/D are linked with a higher-than-average blood pressure level^46^. Women who were homozygous for the *AGT* M235T M variant had significantly higher plasma angiotensinogen concentrations than those who were homozygous for the T variant, which might represent a possible pathogenic mechanism of PE^18^.

Few studies have examined the fetal genetic effects of *RAS* genes on maternal PE risk. A previous Romanian case-control study included 36 mother/newborn pairs with PE complications and 71 controls. Their findings on fetal *ACE* G2350A, *AGT* 174M, and *ACE* I/D were in accordance with our results^21^. The discrepancy in the effect of fetal *AGT* M235T, which was positive in the current study but negative in the Romanian study, may partly arise from the different study designs, besides the different genetic background and sample size. Moreover, Arngrimsson *et al*. demonstrated that the *AGT* M235T variant might be of paternal origin^47^ and Takimoto *et al*. also demonstrated that a single renin gene inherited from the father in newborn mice might increase the risk of maternal hypertension during pregnancy^17^. The study of Walther T found ACE activity was significantly higher in normal fetuses than incorresponding maternal plasma^16^, which may suggest a more evident function of the fetal ACE gene than the maternal gene.

Many previous studies have investigated the association between the five maternal genotypes and PE, but the conclusions were weak and inconsistent^21, 35, 48, 49^. The maternal *ACE* I/D, *AGT* M235T, *AGT* T174M, and *AT1R* A1166C genotypes were not associated with PE in our study, which is consistent with the results of the recently published meta-analyses^50–52^. Few studies have investigated the effect of maternal *ACE* G2350A in the Asian population. The higher power of our study may provide additional evidence to corroborate the negative associations.

To test the interaction between maternal genes and environmental factors, we mainly considered education background and pre-pregnancy BMI. Screening of risk factors revealed that the education status of the mothers might influence their health behaviors and nutrition^53^, which may in turn play a role in PE development. However, the LRT found no interaction between the education background and gene polymorphisms. With regard to the interaction of BMI and *AGT* M235T, a previous Japanese case-control study showed a valid interaction^29^. In our study, while a significant association was suggested by the LRT test, the association was not significant in the subsequent stratified analysis, which may be due to the limited power of the decreased sample size after stratification.

The current study demonstrated, for the first time, that fetal *ACE* I/D, *ACE* G2350A, *AGT* M235T, and *AT1R* A1166C were significantly associated with PE development in Han Chinese women. Further, an analysis of gene–environmental interactions revealed that a pre-pregnancy BMI > 24 was likely to interact with *AGT* M235T and *AGT* T174M in PE pathogenesis. Although we performed in-depth analyses to explore maternal/fetal genetic effects on PE, early PE, as a special disease entity of PE, was not investigated. Besides, as important indicators of the liver function and outcome predictors of PE patients^54, 55^, the association between PE and the level of aspartate aminotransferase (AST), and alanine aminotransferase (ALT) were also not analyzed. Nevertheless, our findings on the fetal SNPs reinforce the view that fetal genes contribute to PE development^19^; further prospective studies are needed to confirm and replicate these associations.

## Methods

### Study Settings and Participants

This was a hybrid study that included case-parent triads and control-mother dyads, recruiting participants from January 2008 to October 2014 from two Maternal and Child Care Hospitals in Hubei and Henan provinces. The study was approved by the Institutional Review Board of Tongji Medical College, and all participants gave informed consent before participating in the study. All experiments were carried out in accordance with relevant guidelines and regulations. PE patients who fulfilled the following criteria were recruited: BP ≥ 140/90 mmHg after 20 weeks of gestation and new onset of proteinuria, with or without convulsions or seizures. sPE was distinguished from mPE, using the following two criteria: BP ≥ 160/110 mmHg on two occasions at least 6 h apart in a woman on bed rest, accompanied by proteinuria ≥3+ reading on dipstick testing on two random samples at least 6 h apart^56^. Controls were healthy normotensive pregnant women delivering at the same hospital. Exclusion criteria included a history of cardiovascular disease, diabetes mellitus, renal disease, or other pregnancy complications, as well as a serious abnormality in the neonate or a multiple gestation. According to the current diagnostic criteria of IUGR in China^57^, all newborns with a birth weight below the tenth percentile or less than two standard deviations below the mean weight for gestational age, or term infants with a birth weight less than 2500 grams can be diagnosed as IUGR. Two hundred and fifty-six PE patients, with their partners and offspring, and 360 controls with their offspring, all of Han ethnicity, were recruited. Among the 256 PE patients, 39 exhibited mPE and 217 exhibited sPE.

### Genotyping Assays

In the case group, we collected 5 mL of venous blood from the patients and their partners, and umbilical cord blood from the fetuses; in the control group, we collected 5 mL of venous blood from the patients and the umbilical cord, in EDTA-containing tubes. Genomic DNA was extracted from blood leukocytes, using the Puregene^®^ Blood Kit (QIAGEN, Germantown, MD, USA), and was stored at −80 °C. DNA quality and quantity was evaluated using a NanoDrop™ 2000 spectrophotometer (Wilmington, DE, USA). The *ACE* I/D genotypes were determined by polymerase chain reaction (PCR) amplification and agarose gel electrophoresis, as described previously^58^. To avoid the mistyping of the ID genotype as DD, each sample found to have the DD genotype was subjected to a second independent PCR amplification with a set of primers that recognize an insertion-specific sequence, designed as described by Lindpaintner *et al*.^59^ (see Supplementary Fig. S1). The remaining four polymorphisms were genotyped using TaqMan™ SNP Genotyping Assays (Applied Biosystems [ABI], Foster City, CA, USA) according to the manufacturer’s protocol, using the 7900HT Fast Real-Time PCR System (ABI).

### Statistical Analysis

In the current study, the most frequently homozygous parental genotypes were regarded as the reference genotypes. A 2-tailed *P*-value < 0.05 was considered statistically significant. HWE for all genotypes in the control group was evaluated by a goodness-of-fit χ^2^ test. Differences in the demographic characteristics and genotype frequency distributions between the cases and controls were evaluated by Pearson’s χ^2^ and Student’s *t*-tests, where appropriate. The Monte Carlo method and Fisher’s exact probability were employed, respectively, instead of Pearson’s χ^2^ test when >20% cells had expected frequencies between 1 and 5 or when any frequency was <1. The low-frequency alleles of each SNP were regarded as mutant type, and the effects of the polymorphisms were evaluated using the dominant (heterozygous + homozygous mutant type *vs*. homozygous wild type) and recessive (homozygous mutant type *vs*. homozygous wild type and heterozygous) models. Family-based association analyses were performed using TDT^39^. A log-linear modeling approach^60^ was implemented to estimate the relative risks (ORs) of the maternal and fetal genotypes. The effects of maternal and fetal gene interactions were analyzed by logistic regression. LRT was implemented to test the gene-environment interaction based on case-parent triad design^43^. When the LRT indicated the presence of an interaction (*P*_LRT_ < 0.05), the ORs and 95% CI were calculated separately for different strata of the exposure variable. Statistical results were adjusted for multiple testing using the FDR procedures reported by Benjamini *et al*.^61^.

Sample size was calculated by a power calculation method based on the noncentrality parameter for a four-df chi-squared LRT^40^. For each of the five SNPs with risk allele frequencies of 0.10–0.36, a sample size of 220 PE cases and 300 controls was estimated to be sufficient to detect an effect size of 1.5 in the log-linear approach, with a power of 0.90 when the significance level (α) was 0.05. LRT was performed using the Log-linear Expectation Maximization (LEM) software (examples of the LEM scripts are available at http://www.niehs.nih.gov/research/resources/software/biostatistics/lem/index.cfm ). Other statistical analyses were performed with SPSS (Version 21.0; SPSS, Chicago, IL, USA).

## Electronic supplementary material

Supplementary Information
