# Supplementary material for: The Gene Variants of Maternal/Fetal Renin-Angiotensin System in Preeclampsia: A Hybrid Case-Parent/Mother-Control Study
Source: Sci Rep. 2017 Jul 11;7:5087. doi: 10.1038/s41598-017-05411-z (PMC5506018; doi:10.1038/s41598-017-05411-z)
Supplement: Supplementary file 1 — Supplementary Information [file 41598_2017_5411_MOESM1_ESM.pdf]

The Gene Variants of Maternal/Fetal Renin-Angiotensin System Gene in Preeclampsia: A Hybrid Case-Parent/ Mother-Control Study

Heng Zhang[1], Ying-Xue Li[1,2], Wei-Jun Peng[1], Zhi-Wei Li[3], Chun-Hua Zhang[4], Hai-Hong Di[3], Xian-Ping Shen[5], Jun-Feng Zhu[6] and Wei-Rong Yan[1*]

Supplementary Table S1. Distributions of the maternal and fetal genotypes and allele frequency of the selected single nucleotide polymorphisms (SNPs) in PE cases and controls

		Maternal		*P*	*P**	Fetal		*P*	*P**
		Case n (%)	Control n (%)			Case n (%)	Control n (%)		
ACE I/D	DD	79 (31.6)	113 (32.2)	0.782	0.997	90 (36.0)	112 (31.9)	0.545	0.997
	ID	119 (47.6)	158 (45.0)			119 (47.6)	174 (49.6)		
	II	52 (20.8)	80 (22.8)			41 (16.4)	65 (18.5)		
	D	277 (55.4)	384 (54.7)	0.810	0.997	299 (59.8)	398 (56.7)	0.282	0.997
	I	223 (44.6)	318 (45.3)			201 (40.2)	304 (43.3)		

	AA	100 (42.8)	153 (44.6)	0.888	0.997	111 (47.4)	157 (45.8)	0.874	0.997
	AG	107 (45.7)	150 (43.7)			103 (44.0)	153 (44.6)		
ACE G2350A	GG	27 (11.5)	40 (11.7)			20 (8.6)	33 (9.6)		
	A	307 (65.6)	456 (66.5)	0.758	0.997	325 (69.4)	467 (68.1)	0.623	0.997
	G	161 (34.4)	230 (33.5)			143 (30.6)	219 (31.9)		
	TT	135 (54.2)	197 (56.8)	0.559	0.997	119 (47.8)	196 (56.5)	0.093	0.930
	MT	96 (38.6)	120 (34.6)			111(44.6)	125 (36.0)		
AGT M235T	MM	18 (7.2)	30 (8.6)			19 (7.6)	26 (7.5)		
	T	366 (73.5)	514 (74.1)	0.825	0.997	349 (70.1)	517 (25.5)	0.092	0.930
	M	132 (26.5)	180 (25.9)			149 (29.9)	177 (74.5)		
	TT	189 (79.4)	277 (79.4)	0.664	0.997	201 (84.4)	283 (81.1)	0.061	0.930
AGT T174M	MT	44 (18.5)	68 (19.5)			33 (13.9)	65 (18.6)		
	MM	5 (2.1)	4 (1.1)			4 (1.7)	1 (0.3)		
	T	422 (88.7)	622 (89.1)	0.807	0.997	435 (91.4)	631 (90.4)	0.566	0.997

	M	54 (11.3)	76 (10.9)			41 (8.6)	67 (9.6)		
AT1R A1166C	AA	206 (87.7)	306 (88.2)	0.955	1.000	213 (90.6)	313 (90.2)	0.711	0.997
	AC	28 (11.9)	39 (11.2)			22 (9.4)	33 (9.5)		
	CC	1 (0.4)	2 (0.6)			0 (0.0)	1 (0.3)		
	A	440 (93.6)	651 (93.8)	0.897	0.997	448 (95.3)	659 (95.4)	0.779	0.997
	C	30 (6.4)	43 (6.2)			22 (4.7)	35 (4.6)		

*P**, corrected *P*-value after false discovery rate (FDR) correction.

Supplementary Table S2. Distributions of the maternal and fetal genotypes the selected single nucleotide polymorphisms (SNPs) in IUGR cases and controls

		Maternal		*P*	*P**	Fetal		*P*	*P**
		Case n (%)	Control n (%)			Case n (%)	Control n (%)		
ACE I/D	DD	23(30.7)	150 (3 3.7)	0.091		32 (42.7)	145 (32.6)	0.033	0.997
	ID	42 (56.0)	195 (43.8)			38 (50.7)	221 (49.7)		
	II	10 (13.3)	100 (22.5)			5 (6.7)	79 (17.8)		
ACE G2350A	AA	26 (36.1)	194 (45.4)	0.313	0.997	34 (47.2)	197 (46.1)	0.567	0.997
	AG	38 (52.8)	187 (43.8)			34 (47.2)	190 (44.5)		
	GG	8 (11.1)	46 (10.8)			4 (5.6)	40 (9.4)		
AGT M235T	TT	47 (62.7)	247 (55.9)	0.548	0.997	38 (50.7)	236 (53.4)	0.093	0.185
	MT	23 (30.7)	160 (36.2)			35(46.7)	172 (38.9)		
	MM	5 (6.7)	35 (7.9)			2 (2.7)	34 (7.7)		
AGT T174M	TT	56(81.2)	345 (78.8)	0.558	0.997	60 (87.0)	358 (81.7)	0.453	0.930

	MT	13(18.8)	86 (19.6)			9 (13.0)	75 (17.1)		
	MM	0 (0.0)	7 (1.6)			0 (0.0)	5 (1.1)		
AT1R A1166C	AA	60 (85.7)	384 (87.5)	0.600	1.000	61 (87.1)	400 (91.1)	0.492	0.492
	AC	9 (12.9)	53 (12.1)			9 (12.9)	38 (8.7)		
	CC	1 (1.4)	2 (0.5)			0 (0.0)	1 (0.2)		

IUGR, intrauterine growth restriction.

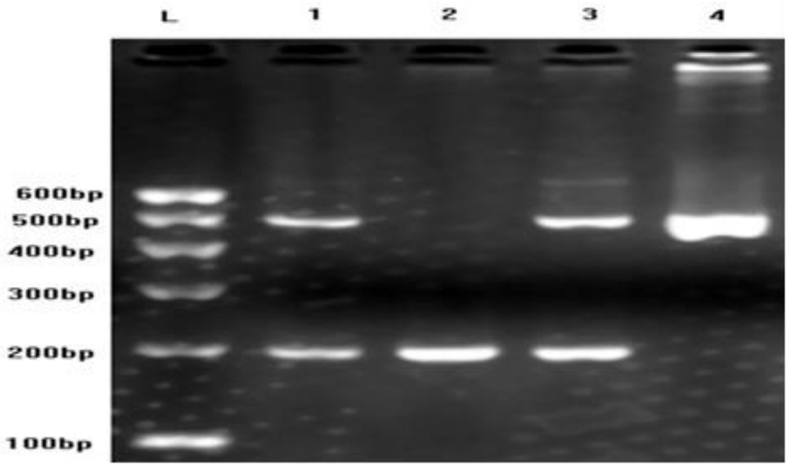

Supplementary Figure S1. Representative electropherogram and analysis of ACE I/D polymorphism
Numbers 1 and 3 show I/D genotype; number 2 shows D/D genotype, number 4 shows I/I genotype.
